# Alzheimer’s disease is associated with disruption in thiamin transport physiology: A potential role for neuroinflammation

**DOI:** 10.1016/j.nbd.2022.105799

**Published:** 2022-06-21

**Authors:** Kalidas Ramamoorthy, Ryan Yoshimura, Saleh Al-Juburi, Kasin Y. Anandam, Rubina Kapadia, Amal Alachkar, Geoffrey W. Abbott, Hamid M. Said

**Affiliations:** a Departments of Physiology & Biophysics, University of California, Irvine, CA 92697, United States of America; b Medicine, School of Medicine, University of California, Irvine, CA 92697, United States of America; c Department of Pharmaceutical Sciences, University of California, Irvine, CA 92697, United States of America; d Department of Veteran Affairs, VA Medical Center, Long Beach, CA 90822, United States of America

**Keywords:** Alzheimer’s disease, Thiamin transporters –1 and –2, Pro-inflammatory cytokines, Thiamin uptake

## Abstract

Alzheimer’s disease (AD) is a neurodegenerative disease characterized by Amyloid-β peptide (Aβ) containing plaques and cognitive deficits. The pathophysiology of AD also involves neuroinflammation. Vitamin B1 (thiamin) is indispensable for normal cellular energy metabolism. Thiamin homeostasis is altered in AD, and its deficiency is known to aggravate AD pathology. Little, however, is known about possible alterations in level of expression of thiamin transporters-1 and -2 (THTR-1 and -2) in the brain of AD, and whether pro-inflammatory cytokines affect thiamin uptake by brain cells. We addressed these issues using brain tissue samples [prefrontal cortex (PFC) and hippocampus (HIP)] from AD patients and from 5XFAD mouse model of AD, together with cultured human neuroblastoma SH-SY5Y cells as model. Our results revealed a significantly lower expression of both THTR-1 and THTR-2 in the PFC and HIP of AD patients and 5XFAD mouse model of AD compared to appropriate normal controls. Further, we found that exposure of the SH-SY5Y cells to pro-inflammatory cytokines (IL-1β, IL-6, and TNF-α) led to a significant inhibition in thiamin uptake. Focusing on IL-1β, we found the inhibition in thiamin uptake to be time-dependent and reversible; it was also associated with a substantial reduction in expression of THTR-1 (but not THTR-2) protein and mRNA as well as a decrease in promoter activity of the *SLC19A2* gene (which encodes THTR-1). Finally, using transcriptomic analysis, we found that thiamin availability in SH-SY5Y cells caused changes in the expression of genes relevant to AD pathways. These studies demonstrate, for the first time, that thiamin transport physiology/molecular biology parameters are negatively impacted in AD brain and that pro-inflammatory cytokines inhibit thiamin uptake by neuroblastoma cells. The results also support a possible role for thiamin in the pathophysiology of AD.

## 1. Introduction

The water-soluble vitamin B1 (thiamin; also referred to as the “energy vitamin”) is indispensable for normal human health and physiology. In its predominant and biologically active form, thiamin pyrophosphate (TPP), the vitamin serves as a cofactor for multiple enzymes (transketolase, pyruvate dehydrogenase, alpha-ketoglutarate dehydrogenase, and branched-chain keto-acid dehydrogenase) that are involved in critical metabolic reactions including oxidative energy metabolism and ATP production (Berdanier, 1998; Martin, 2001; Tanphaichitr, 1994). Thiamin also has anti-inflammatory effects (Sambon et al., 2021) and plays a vital role in reducing cellular oxidative stress (Calingasan et al., 1999; Portari et al., 2008; Martin, 2001; Todd and Butterworth, 1997). Thus, deficiency of thiamin at the cellular level leads to impairment in oxidative energy metabolism/decrease in cellular ATP level in neuronal and other cell-types (Aikawa et al., 1984; Bettendorff and Wins, 1994; Butterworth, 2003; Hernandez-Vazquez et al., 2016), and a propensity for oxidative stress (Hazell and Butterworth, 2009); it also leads to impairment in the function/structure of mitochondria (an organelle that contains/utilizes ~90% of cellular TPP) (Bettendorff et al., 1995). At the systemic level, thiamin deficiency leads to various clinical abnormalities, including neurological disorders. The deleterious role of thiamin deficiency in nervous system abnormalities of Wernicke-Korsakoff syndrome and beriberi is well established (Pongpanich et al., 1974; Yellowlees, 1986). Also, mutations in thiamin transporters and metabolism genes *SLC25A19, SLC19A3* and *TPK1* have been causally linked with Leigh syndrome, an early-in-life progressive neurodegenerative disorder (Mayr et al., 2011; Liu et al., 2017b; Plaitakis et al., 1980; Zeng et al., 2005).

Like all human/mammalian cells, brain cells do not synthesize thiamin and thus, need to obtain it from blood circulation. Two specific transporters mediate the uptake of free thiamin across the plasma membrane of mammalian cells: thiamin transporter- 1 and – 2 (THTR-1 and THTR-2, products of the *SLC19A2* and *SLC19A3* genes, respectively). These transporters have different affinities (apparent Km) for thiamin; it is in the micromolar range for THTR-1 and in the nanomolar range for THTR-2 (Dutta et al., 1999; Said et al., 2004). In addition, level of expression of these transporters varies in different cell-types (Dutta et al., 1999). Once internalized, the majority of the free thiamin (85 to 90%) is then enzymatically converted to TPP in the cytoplasm, with the majority of the latter then taken up by the mitochondria for utilization in a variety of metabolic reactions related to oxidative energy metabolism and ATP production (Bettendorff, 1994; Bettendorff et al., 1994; Bettendorff, 1995).

Alzheimer’s disease (AD) is a neurodegenerative disease characterized by amyloid-β peptide (Aβ)- containing plaques, neurofibrillary tangles, and cognitive deficits (Masters et al., 1985; Mucke and Selkoe, 2012). Mitochondrial dysfunction, oxidative stress, and diminished glucose metabolism are consistent features of AD that occur early in the disease preceding the β*β*-amyloid and tau pathology. Therefore, these changes may be causally linked to the pathogenesis of the disease and the formation of plaques and tangles (Manczak et al., 2006; Moreira et al., 2006; Ohta and Ohsawa, 2006; Oka et al., 2016; Völgyi et al., 2018). Neuroinflammation occurs over the degenerative course of the disease and leads to progressive synapse loss and degeneration of specific neuronal populations (Baleriola et al., 2014; Monsonego et al., 2001; Selkoe, 2002; Ferreira and Klein, 2011; Ferreira et al., 2014; Lue et al., 2001). AD pathophysiology also includes neuroinflammation, which is associated with elevation of pro-inflammatory factors such as tumor necrosis factor alpha α (TNF-α), interleukin 1β (IL-1β), interleukin 6 (IL-6), and other cytokines population (Italiani et al., 2018; Zhao et al., 2003; Zuliani et al., 2007).

Compelling evidence suggests that thiamin homeostasis might also be disrupted in AD. Thiamin diphosphate (TDP) levels decrease, abnormal activities of TDP-dependent key enzymes and glucose metabolism reduction have been reported in the blood and brain of AD patients (Gibson et al., 1988; Héroux et al., 1996; Mastrogiacomo et al., 1996). In addition, many thiamin-dependent processes are diminished in the brains of AD patients (Gibson et al., 2013). For example, reduced brain glucose metabolism and increased oxidative stress consistently occur in both AD and thiamin deficiency; TDP level reduction in AD strongly correlates with reduced brain glucose hypometabolism (Sang et al., 2018). Thiamin deficiency aggravates plaque formation and alters the metabolism of Amyloid Precursor Protein Processing (APP) and/or Aβ in mouse models of AD (Karuppagounder et al., 2009).

There is little known about possible alteration in the level of expression of THTR-1 and -2 in brain tissue of AD patients. Also, while recent studies have shown that chronic exposure to pro-inflammatory cytokines inhibits thiamin uptake by non-brain cell types (Anandam et al., 2021; Zhu et al., 2015), it is not known whether similar effects can be observed in neuronal cells. In this work, we approached these questions comprehensively and have utilized appropriate human and animal brain tissue samples as well as a proper in vitro cell culture model. We first examined the changes in the expression of THTR-1 and THTR-2 in the prefrontal cortex (PFC) and hippocampus (HIP) tissues of AD patients and (5XFAD) mouse model of AD. We also utilized the human neuroblastoma cells SH-SY5Y as a model to examine the effect of prolonged exposure to pro-inflammatory cytokines (especially those whose levels are increased in AD, i.e., IL-1β, IL-6, and TNF-α) on thiamin uptake and the possible molecular mechanisms. Finally, we used transcriptome analysis to examine the effect of thiamin availability on gene expression profile in SH-SY5Y cells.

## 2. Materials and methods

### 2.1. Materials

The following materials and agents were obtained from commercial sources: Human neuroblastoma cell line SH-SY5Y was acquired from ATCC (Rockville, MD); [^3^H] Thiamin (specific activity 12.8 Ci/mmol; radiochemical purity of 98%) from Moravek (Brea, CA); nitrocellulose filters (0.45 mm) from Millipore (Fisher Scientific); human IL-1β, IL-6 from R&D Biosystems (Minneapolis, MN); TNF-α from Gibco (Fisher Scientific); Anti-THTR-1 (ab123246) from Abcam (Cambridge, MA); anti-Sp-1 (07645) from Millipore (Billerica, MA); anti-β-actin (sc-47778) from Santa Cruz Biotechnology (Santa Cruz, CA); and secondary antibodies anti-rabbit IRDye-800 and anti-mouse IRDye-680 from LI-COR Bioscience (Lincoln, NE). Oligonucleotide primers were synthesized by Integrated DNA Technologies (San Diego, CA). All chemicals such as unlabeled thiamin and molecular biology reagents were of analytical grade and obtained from commercial sources.

### 2.2. Human and mice brain tissue collection

Human brain tissue samples of AD patients and their age-comparable controls were obtained from cadaveric brain tissues of organ donors received from three centers: Mount Sinai-NBTR (NeuroBioBank Brain and Tissue Repositories); NY, USA; The UC Irvine Institute for Memory Impairments and Neurological Disorders (UCI-MIND), Irvine, CA, USA; and The National Disease Research Interchange (NDRI), PA, USA]. The frozen prefrontal cortex (PFC) and hippocampus (HIP) tissue samples were received (24–48 h post isolation) and used in our investigations (using a University of California, Irvine approved protocol; Institutional Review Board: 2017–1593). Mice [Wild-Type and 5XFAD (AD animal model; Oblak et al., 2021)] of both sexes (8 weeks older) were used in our studies and were obtained from UC Irvine Transgenic Mouse Facility (UCI-TMF), CA, UCI. The PFC and HIP brain regions were surgically removed from mice following a protocol approved by the Institutional Animal Care and Use Committee of the University of California, Irvine, CA. Briefly, the mice were euthanized by inhalation of carbon dioxide (CO_2_). The brains were rapidly removed and frozen in isopentane at approximately −40 °C and stored at −80 °C. 500 μm coronal sections of the brains were cut, using a cryostat, at the levels of the PFC and HIP. Tissues from the PFC and HIP from both hemispheres were excised using 2 mm stainless steel biopsy punch needles.

### 2.3. Cell culture, exposure to cytokines, and carrier-mediated [^3^H] thiamin uptake

Human neuroblastoma SH-SY5Y cells were cultured in Dulbecco’s Modified Eagle’s Medium (DMEM) supplemented with FBS (10%), penicillin/streptomycin (100 U/mL) in a humidified environment at 37 °C with 5% CO_2_ incubator (Miao et al., 2020; Zhang et al., 2020). After reaching 80% confluency, the cells were exposed to the pro-inflammatory cytokines IL-1β (50 ng/mL), IL-6 (50 ng/mL) and TNF-α (50 ng/mL) (Brosseron et al., 2014; Veerhuis et al., 1999) for 48 h (unless otherwise stated), as described previously (Anandam et al., 2021). [^3^H] Thiamin uptake (15 nM; 10 min) was then examined at 37 °C in Krebs–Ringer (KR) buffer in the presence and absence of 1 mM unlabeled thiamin. Uptake was terminated by adding 1 mL of ice-cold KR buffer, followed by rinsing of the cells (with ice-cold KR buffer), digesting them (with 1 mL of 1 N NaOH and then neutralized with 10 N HCl), and counting for radioactive content using a liquid scintillation counter (Anandam et al., 2021; Zhu et al., 2015). Protein contents of the digested cells were measured using the Dc protein kit (Bio-Rad).

### 2.4. Quantitative real-time PCR analysis

Total RNAs were isolated from human and mouse PFC and HIP tissues and from SH-SY5Y cells using Qiazol and the RNeasy Kit (Qiagen, Hilden Germany), then reverse transcribed using the Verso-cDNA Synthesis Kit (Thermo Fisher Scientific). The newly synthesized cDNA samples, together with gene-specific primers (Table 1) were then used in the RT-qPCR as described by us previously (Anandam et al., 2021). Expression of THTR-1 and -2 mRNAs were normalized relative to β-actin and quantified using a relative relationship method (Livak and Schmittgen, 2001).

### 2.5. Transfection of SH-SY5Y cells with SLC19A2 minimal-promoter and firefly luciferase assay

Transient transfection of SH-SY5Y cells with the *SLC19A2* minimal promoter-luciferase reporter construct (previously generated and characterized in our laboratory; Reidling and Said, 2003) was performed using Lipofectamine 2000. Transfected cells were then exposed to IL-1β (50 ng/ mL; 48 h) and lysed with passive lysis buffer, followed by determination of luciferase activity using a dual-luciferase assay system (Promega).

### 2.6. Protein isolation and western blot analysis

Control and IL-1β (50 ng/mL)-treated SH-SY5Y cells were lysed in radioimmunoprecipitation assay buffer (Sigma) in the presence of a protease inhibitor cocktail (Roche, Branchburg, NJ). The soluble protein fraction was collected after centrifugation (14,000 rpm, 20 min) and quantified using the Dc protein kit (Bio-Rad). An equal amount of protein (40 μg) was loaded onto 4–12% bis-Tris gradient mini gels (Invitrogen) and transferred onto Immobilon polyvinylidene difluoride membrane (Fisher Scientific). The blots were then probed with anti-THTR-1 (1:1000), anti-Sp1 (1:500), and anti-β-actin (1:3000). The specificity of the THTR-1 polyclonal antibodies has been validated by us in previous studies (Ramamoorthy et al., 2020). The immune-reactive bands were detected with the corresponding secondary antibodies anti-rabbit IRDye-800 and anti-mouse IRDye-680 (both at 1: 30,000 dilution). The densities of the immune-reactive bands were quantified using LI-COR software (LI-COR Bioscience, Lincoln, NE).

### 2.7. RNA isolation and transcriptome analysis

The SH-SY5Y cells were maintained under vitamin B1-deficient and over-supplemented (100 μM) conditions for 21 days. This was carried out as described by us previously (Ashokkumar et al., 2006). Briefly, cells were maintained in a custom-made thiamin-deficient (no added thiamin) or over-supplemented (100 μM thiamin) DMEM growth media that was supplemented with 2.5% of dialyzed FBS (Gemini Bio). Total RNA was extracted following the manufacturer’s protocol using a RNeasy mini kit (Qiagen, Hilden, Germany). Isolated total RNA of the deficient and over-supplement (OS) groups was used to perform whole-transcript transcriptomics using a GeneAtlas microarray system (Life Technologies, Carlsbad, CA, USA). Sense-strand DNA targets for the entire transcriptome were amplified and biotinylated from the total RNA using a GeneChip WT PLUS reagent kit (Life Technologies, Carlsbad, CA, USA). The ss-cDNA products were then hybridized into Human Gene 2.1 ST array strips (Life Technologies, Carlsbad, CA, USA). Each array contains more than 1.35 million probes for more than 33,500 coding transcripts and more than 11,000 long intergenic non-coding transcripts, with a median of 21 unique probes per transcript. The array strips were then washed, stained, and visualized using the GeneAtlas microarray system. Statistical analysis was performed in the Transcriptome Analysis Console 4.0 (Life Technologies, Carlsbad, CA, USA). A gene-level false discovery rate (FDR) calculation was used to filter false positives (Storey and Tibshirani, 2003). A comparison between the two groups (thiamin-deficient and thiamin-over-supplement) was performed with an FDR *P* < 0.05.

### 2.8. Statistical analysis

All graphs were made using GraphPad Prism 8 software (v. 8, GraphPad Software Inc., La Jolla). Data represented in the graphs are expressed as a percentage relative to simultaneously performed controls. All [^3^H] thiamin (15 nM) carrier-mediated uptake [determined by subtracting uptake in the presence of a high pharmacological concentration of unlabeled thiamin (1 mM) from uptake in its absence], western blotting, RT-qPCR, and luciferase assays were performed on 3–6 separate occasions (independent experiments). Data for western blots and all RT-qPCR were normalized relative to the internal control β-actin. The level of significance was set at *P* < 0.05, and statistical analysis was performed by the Student’s *t*-test or one-way ANOVA.

## 3. Results

### 3.1. Human studies

#### 3.1.1. THTR-1 mRNA levels are higher than that of THTR-2 in normal human brains, and both are decreased in AD

**Fig. 1.**
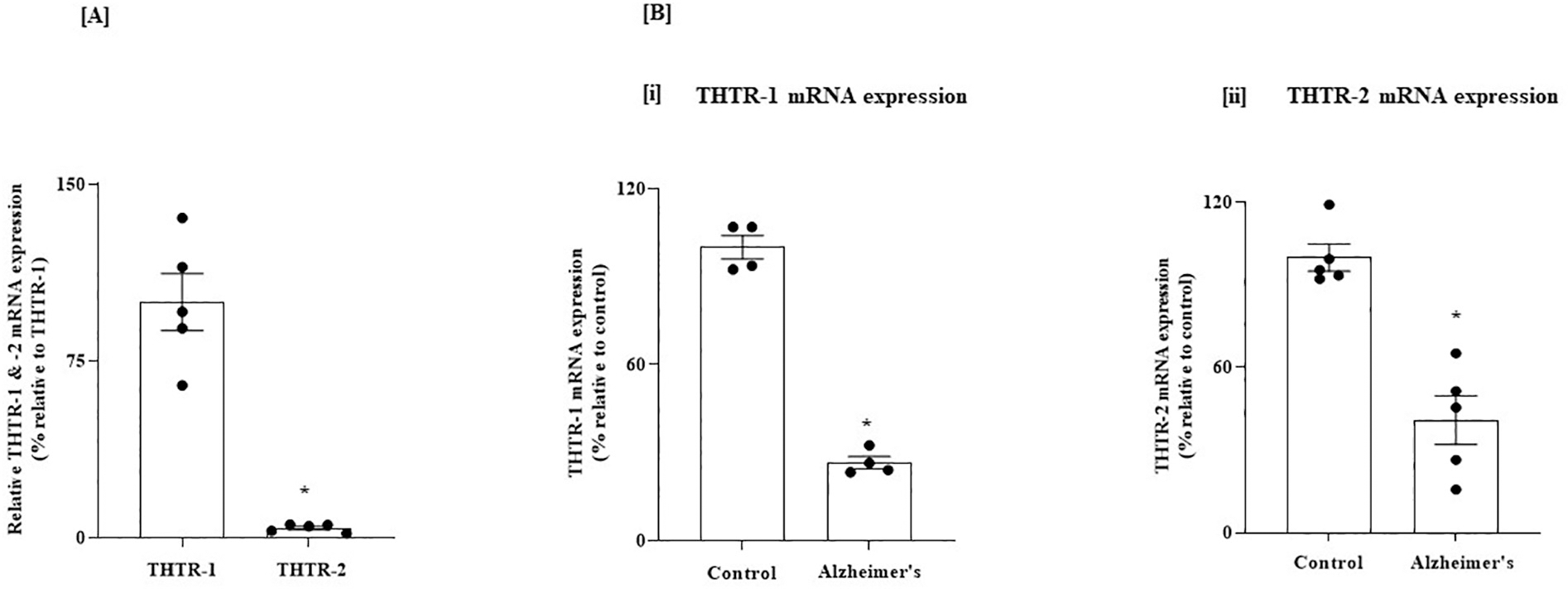
(A) Relative level of expression of THTR-1 and -2 in normal human PFC; and (B) Level of expression of THTR-1 and -2 in PFC of AD and control subjects. (A) PFC tissues from 5 normal subjects were used. (B) PFC tissues from 4 to 5 AD patients and control subjects were used. Level of mRNA expression of THTR-1 (*n* = 4) and THTR-2 (*n* = 5) were determined by mean of RT-qPCR; Data were normalized relative to β actin. Statistical analysis was performed using the Student’s *t*-test. *P < 0.01.

**Fig. 2.**
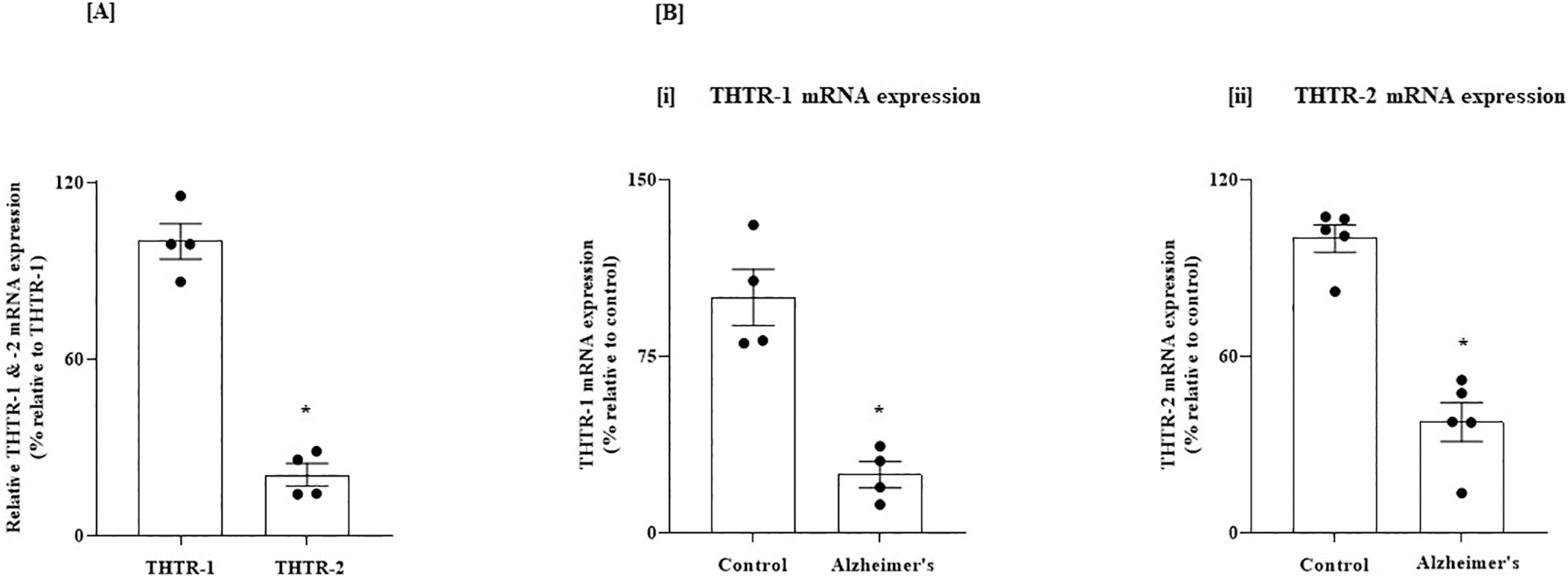
(A) Relative level of expression of THTR-1 and -2 in normal human HIP; and (B) Level of expression of THTR-1 and -2 in HIP of AD and control subjects. (A) HIP tissues from 4 normal subjects were used. (B) HIP tissues from 4 to 5 AD patients and control subjects were used. Level of mRNA expression of THTR-1 (n = 4) and THTR-2 (n = 5) were determined by mean of RT-qPCR. Data were normalized relative to β actin. Statistical analysis was performed using the Student’s *t*-test. *P < 0.01.

We first determined, using RT-qPCR, the relative levels of expressions of THTR-1 and THTR-2 in two normal human brain regions, the PFC and HIP, known to be impacted in AD. The results showed that in both brain regions, THTR-1 mRNA levels are significantly higher than that of THTR-2 (*P* < 0.01 for both Figs. 1A and 2A). We then examined whether the level of THTR-1 and -2 mRNAs are changed in the PFC and HIP of AD patients. The results showed a statistically significant decrease in the expression of THTR-1 and THTR-2 mRNAs (*P* < 0.01 for both) in these two brain regions of AD patients compared to control subjects (Figs. 1B and 2B).

### 3.2. Animal studies

#### 3.2.1. THTR-1 and THTR-2 mRNA levels are decreased in the brains of the 5XFAD mouse model of AD

**Fig. 3.**
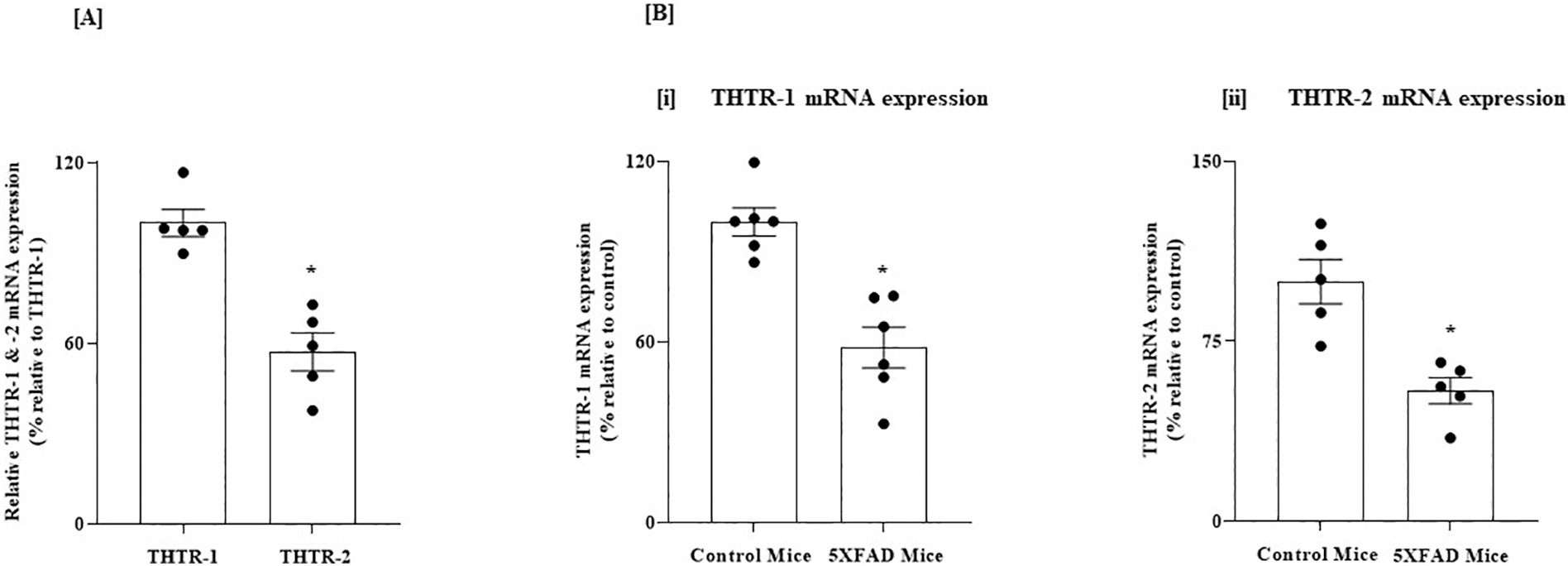
(A) Relative level of expression of THTR-1 and -2 in PFC of normal mice; and (B) Level of expression of THTR-1 and -2 in PFC of 5XFAD mice and WT controls. (A) PFC tissues from 5 WT mice were used. (B) PFC tissues from 5 to 6 5XFAD and WT mice were used. Level of mRNA expression of THTR-1 (*n* = 6) and (ii) THTR-2 (n = 5) in PFC tissues of 5XFAD and WT mice were determined by mean of RT-qPCR. Data were normalized relative to β actin. Statistical analysis was performed using the Student’s *t*-test. *P < 0.01.

**Fig. 4.**
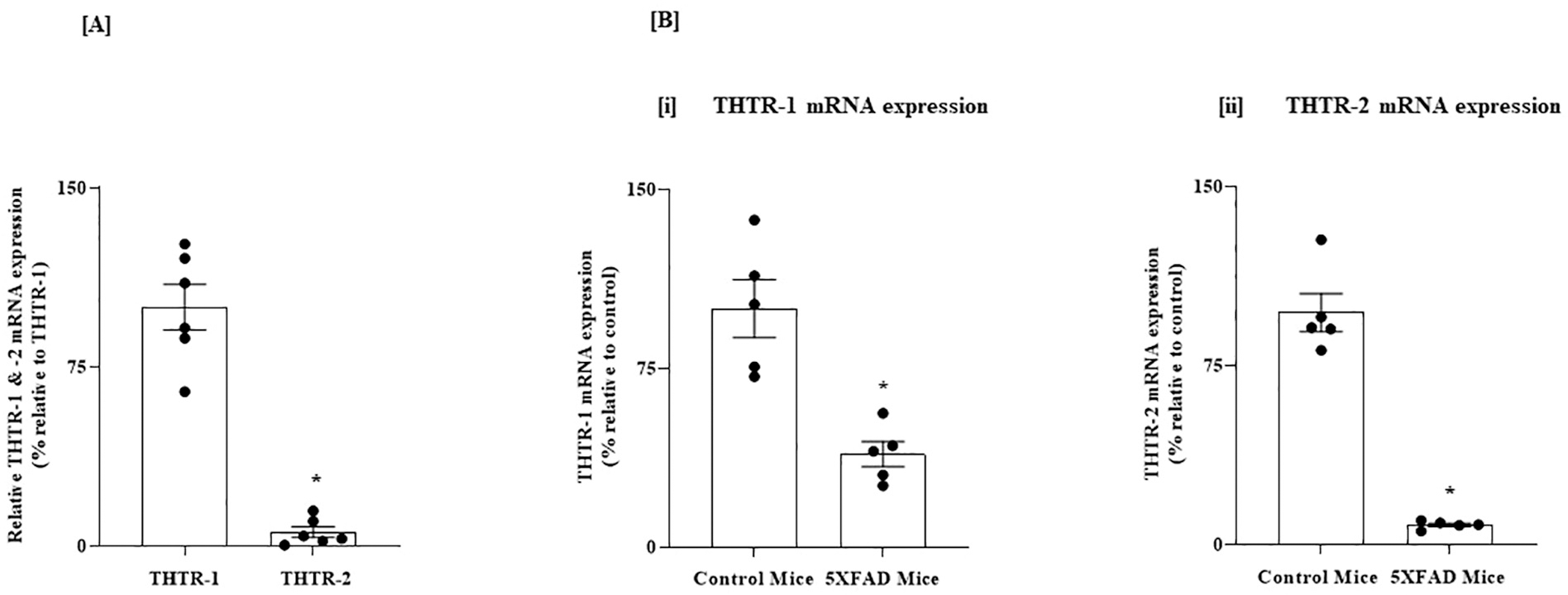
(A) Relative level of expression of THTR-1 and -2 in HIP of normal mice; and (B) Level of expression of THTR-1 and -2 in HIP of 5XFAD mice and WT controls. (A) HIP tissues from 6 WT mice were used. (B) HIP tissues from 5XFAD and WT mice were used. Level of mRNA expression of (i) THTR-1 (n = 5) and (ii) THTR-2 (n = 5) in PC tissue of 5XFAD and WT mice were determined by mean of RT-qPCR. Data were normalized relative to β actin. Statistical analysis was performed using the Student’s *t*-test. *P < 0.01.

In these studies, we first determined (using RT-qPCR) the relative expression of THTR-1 and THTR-2 in PFC and HIP regions of the normal mouse brain. Similar to the human brain, THTR-1 expression was found to be higher than that of THTR-2 in both brain regions (*P* < 0.01 for both Figs. 3A and 4A).

We then examined whether the level of expression of THTR-1 and -2 in PFC and HIP of the 5XFAD mouse model of AD is altered compared to control normal mice (Oblak et al., 2021). Similar to the results in the human brain, there were statistically significant decreases in the mRNA levels of both THTR-1 and THTR-2 in brain samples of the 5XFAD mice compared to wild-type animals (*P* < 0.01 for both, Figs. 3B and 4B).

### 3.3. In vitro studies

#### 3.3.1. Exposure of human SH-SY5Y cells to pro-inflammatory cytokines inhibits thiamin uptake and suppresses expression of its transporters

**Fig. 5.**
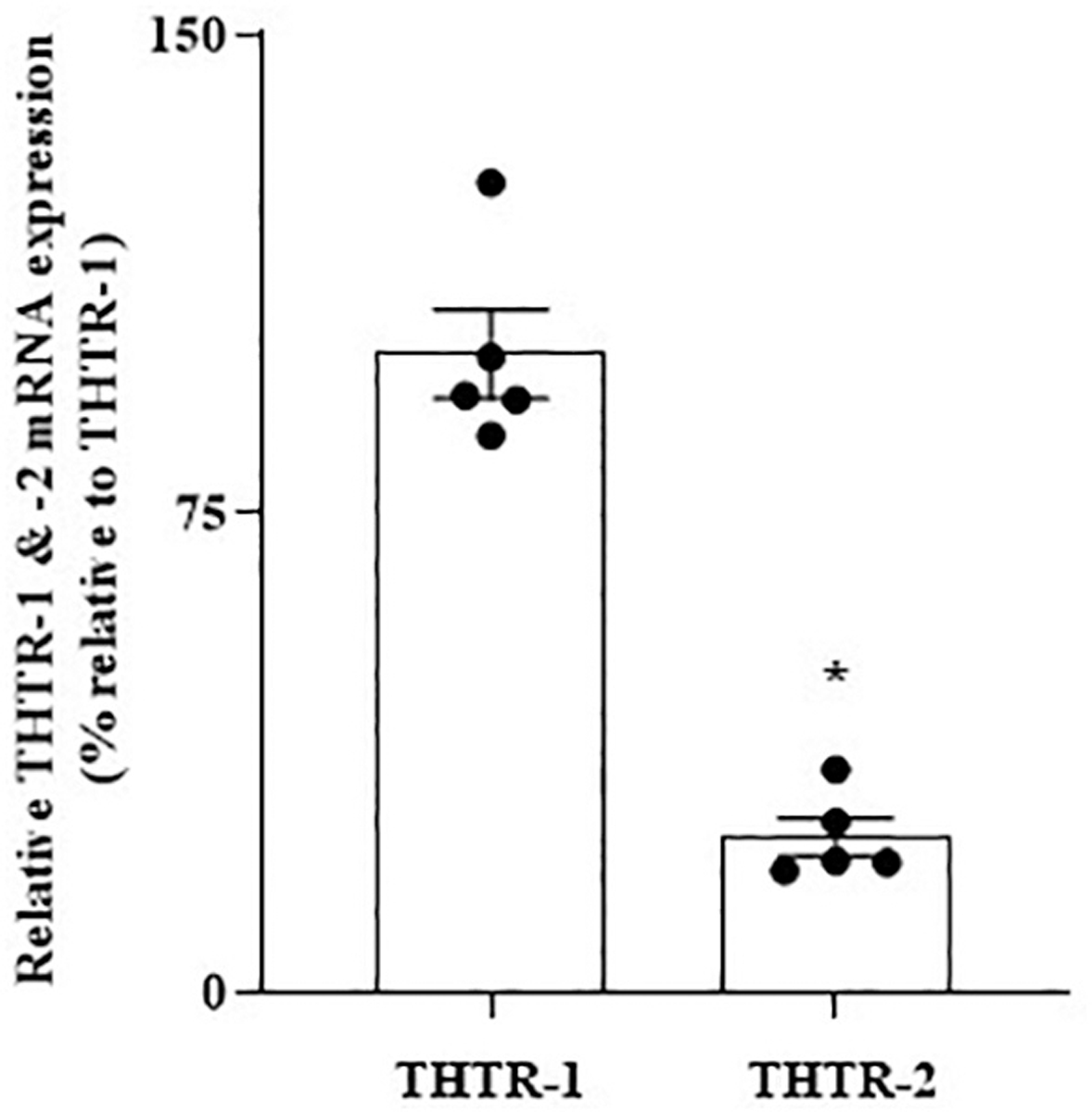
Relative expression of THTR-1 and -2 mRNAs in SH-SY5Y cells. mRNA levels were determined by mean of RT-qPCR (n = 5). Data were normalized relative to β actin. Statistical analysis was performed using the Student’s *t*-test. *P < 0.01.

AD pathophysiology involves neuroinflammation associated with elevated levels of pro-inflammatory cytokines like IL-1β, TNF-α, and IL-6 (Italiani et al., 2018; Zhao et al., 2003; Zuliani et al., 2007). Since prolonged exposure of non-brain cells to such cytokines negatively impacts their ability to take up thiamin (Anandam et al., 2021), we examined here whether such exposure also affects thiamin uptake by brain cells. We tested the effect of exposing human neuroblastoma SH-SY5Y cells to IL-1β, TNF-α, and IL-6 (Brosseron et al., 2014) on thiamin uptake. We first characterized thiamin uptake by these cells by examining the effect of unlabeled thiamin (1 mM) on the initial rate of [^3^H] thiamin (15 nM) uptake. The results showed significant (*P* < 0.01) inhibition of [^3^H] thiamin uptake in the presence of unlabeled thiamin compared to its absence (92.9 ± 3.06 and 38.2 ± 2.74 pmol·mg protein^−1^·10 min^−1^ for control and in the presence of unlabeled thiamin, respectively), indicating that the uptake process was carrier-mediated in nature. Our analysis of the expression of THTR-1 and THTR-2 in the SH-SY5Y cells showed that both transporters are expressed in these cells, with a higher expression of THTR-1 compared to THTR-2 (Fig. 5).

##### 3.3.1.1. Effect of pro-inflammatory cytokines on thiamin uptake and on the expression of THTR-1 and 2.

**Fig. 6.**
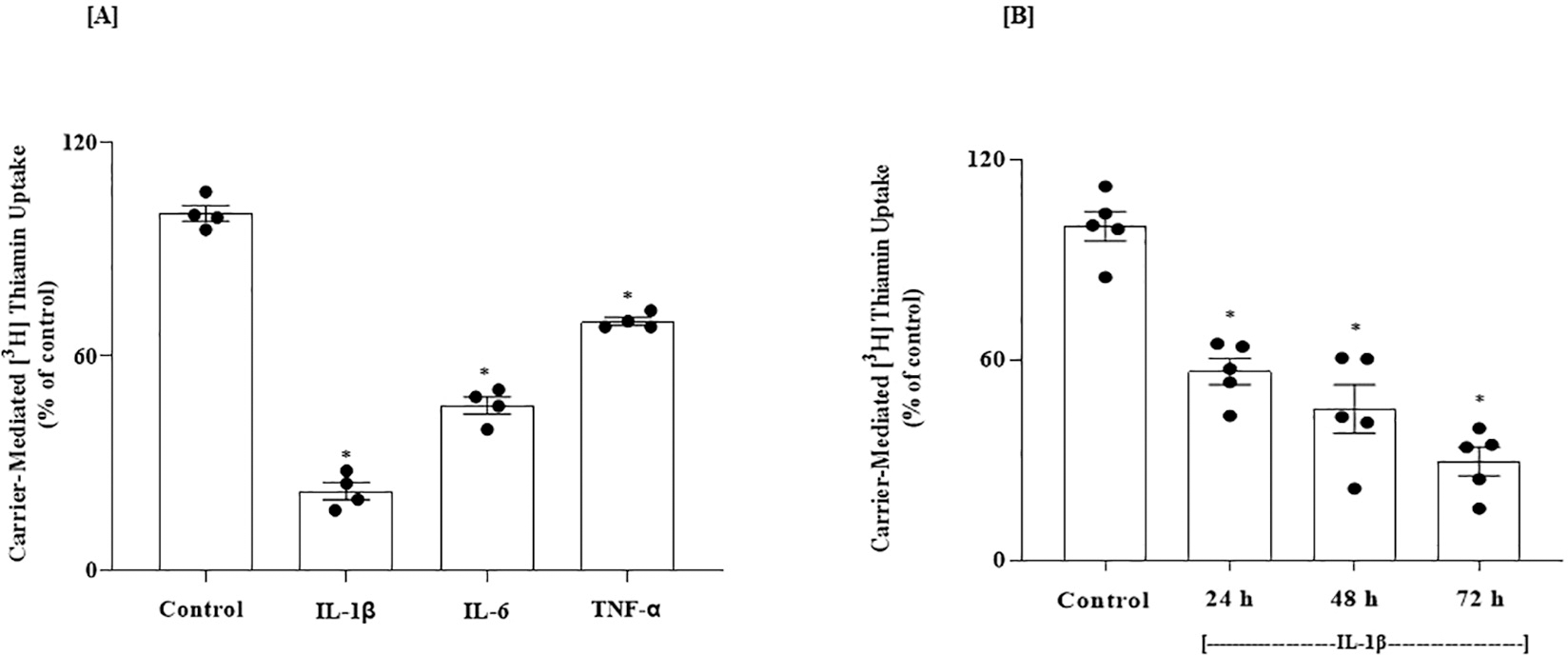
(A) Effect of pro-inflammatory cytokines on carrier-mediated thiamin uptake by SH-SY5Y cells; and (B) Effect of exposure time to IL-1β on thiamin uptake. (A) Cells were treated with IL-1β (50 ng/mL), IL-6 (50 ng/mL) and TNF-α (50 ng/mL)] for 48 h followed by examination of carrier-mediated thiamin (15 nM; 10 min) uptake (n = 4). (B) Cells were treated with IL-1β (50 ng/mL) for different periods of time (24, 48 and 72 h) followed by determination of carrier-mediated thiamin uptake (n = 5). Statistical analysis was performed using one-way ANOVA. *P < 0.01.

We then examined the effect of IL-1β, IL-6, and TNF-α on the initial rate of carrier-mediated thiamin uptake. Our focus on these pro-inflammatory cytokines was based on the fact that their levels are elevated in AD and dementia (Italiani et al., 2018; Veerhuis et al., 1999; Zhao et al., 2003; Zuliani et al., 2007). In these studies, we treated (for 48 h) SH-SY5Y cells with IL-1β (50 ng/mL), IL-6 (50 ng/mL), and TNF-α (50 ng/mL), and the uptake of [^3^H] thiamin (15 nM) was then determined in treated and untreated (control) cells. SH-SY5Y cells treated with all these pro-inflammatory cytokines displayed a significant inhibition in carrier-mediated thiamin uptake compared to untreated controls (*P* < 0.01 for all, Fig. 6A). We also examined the effect of exposure time (24, 48, and 72 h) of the SH-SY5Y cells to the cytokine on the initial rate of [^3^H] thiamin uptake, using IL-1β as a model since it caused the highest degree of inhibition. The results showed a progressive increase in the degree of inhibition in carrier-mediated thiamin uptake as a function of exposure time to IL-1β (Fig. 6B).

**Fig. 7.**
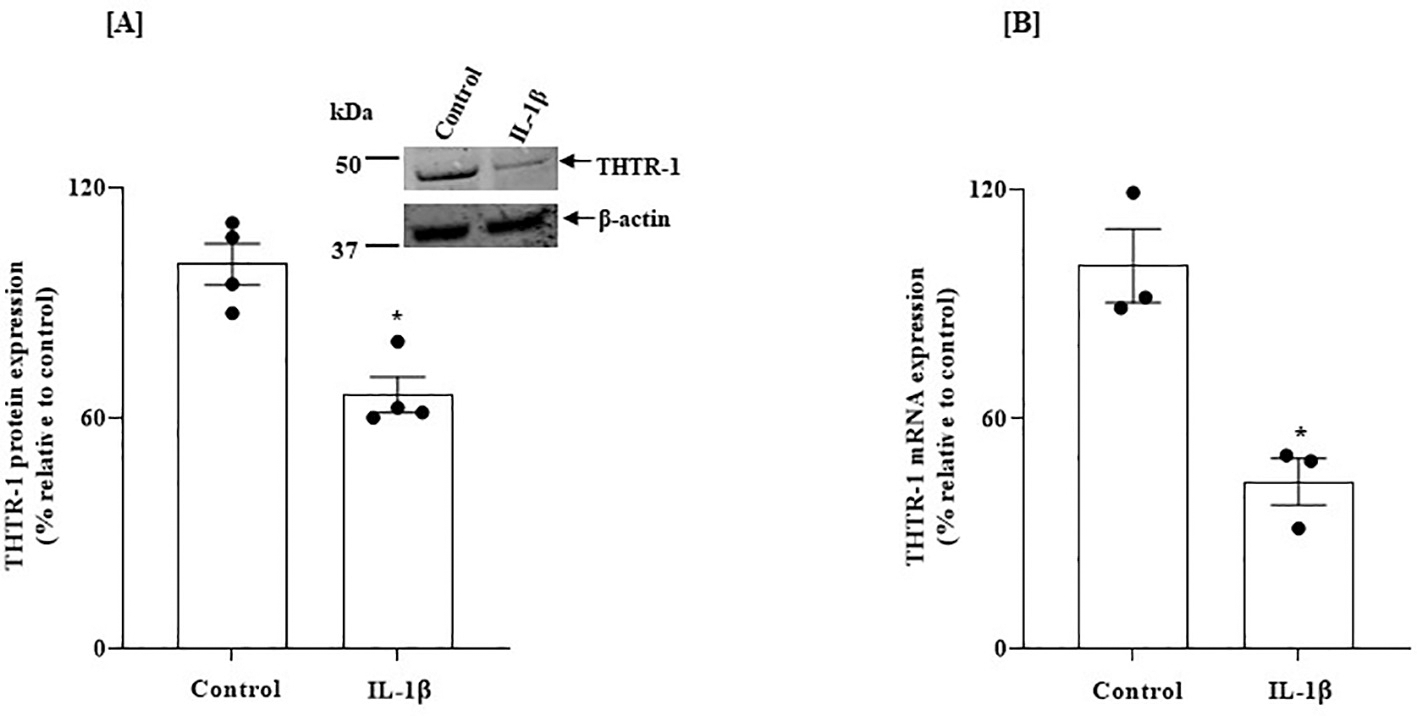
Effect of exposure of SH-SY5Y cells to IL-1β on: (A) level of expression of THTR-1 protein; and on (B) level of THTR-1 mRNA. SH-SY5Y cells were treated with IL-1β (50 ng/mL: 48 h). (A) Western blotting (n = 4) was done using specific anti- THTR-1 antibodies. (B) Level of expression of THTR-1 mRNA was determined by RT-qPCR (for THTR-1; *n* = 3). Protein and mRNA results were normalized relative to β-actin. Statistical analysis was performed using the Student’s *t*-test. *P < 0.01.

In another experiment, we examined the effect of exposure of SH-SY5Y cells to IL-1β on the levels of protein and mRNA of the predominant THTR-1, using western blotting and RT-qPCR. IL-1β -treated cells exhibited reductions in THTR-1 protein and mRNA compared to untreated controls (*P* < 0.01 for both, Fig. 7A and B) [note: expression of THTR-2 was not affected by IL-1β; data not shown].

##### 3.3.1.2. IL-1β effects on thiamin uptake and THTR-1 expression are reversible.

**Fig. 8.**
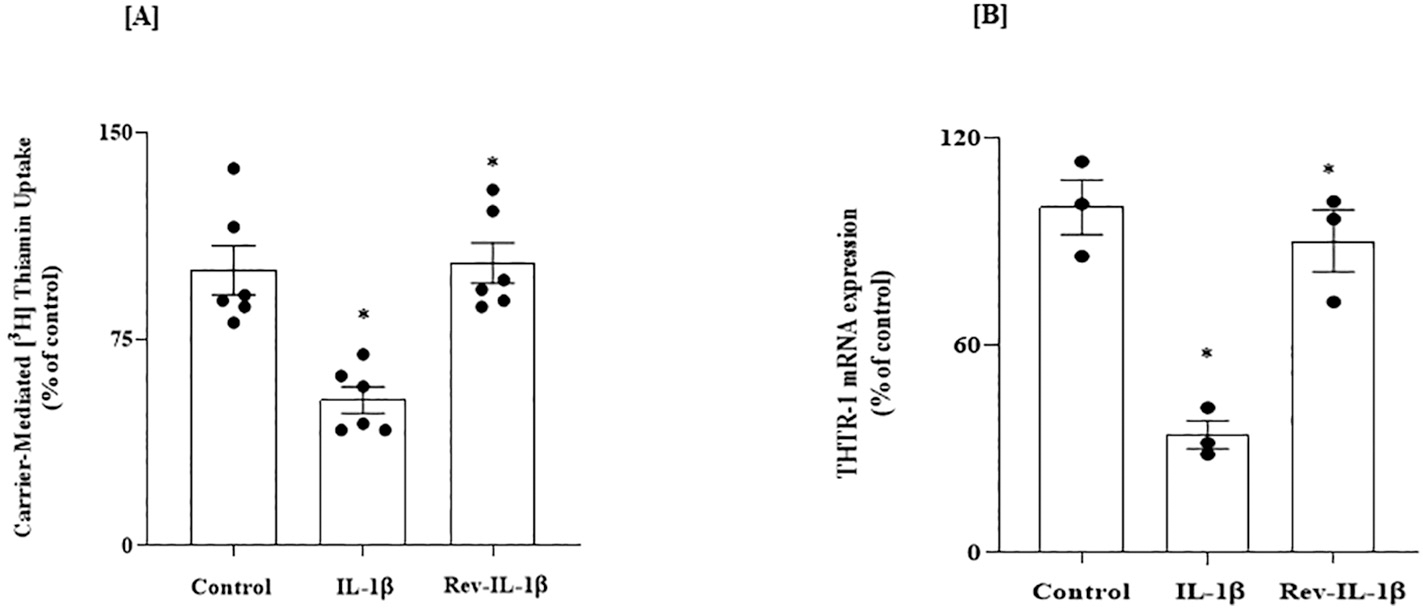
Reversibility of the inhibitory effect of IL-1β on: (A) carrier-mediated thiamin uptake; and (B) level of expression of THTR-1 mRNA, in SH-SY5Y cells. Cells were exposed to IL-1β (48 h) followed by culturing cells in the absence of the cytokine for 24 h. (A) Carrier-mediated uptake was performed (n = 6). (B) Level of expression of THTR-1 mRNA was determined by RT-qPCR (n = 3) and data were normalized relative to β-actin. Statistical analysis was performed using one-way ANOVA. *P < 0.01.

To determine whether IL-1β effects (inhibiting carrier-mediated thiamin uptake and decreasing THTR-1 expression) are reversible, SH-SY5Y cells were treated with IL-1β (50 ng/mL) for 48 h, followed by removal of the pro-inflammatory cytokine for 24 h. The results showed a complete correction of thiamin uptake and an increase of THTR-1 mRNA to normal levels upon removing the pro-inflammatory cytokine to the levels observed in the untreated controls cells (Fig. 8A and B).

##### 3.3.1.3. Transcriptional mechanism(s) mediates the inhibitory effect of IL-1β on THTR-1 expression.

**Fig. 9.**
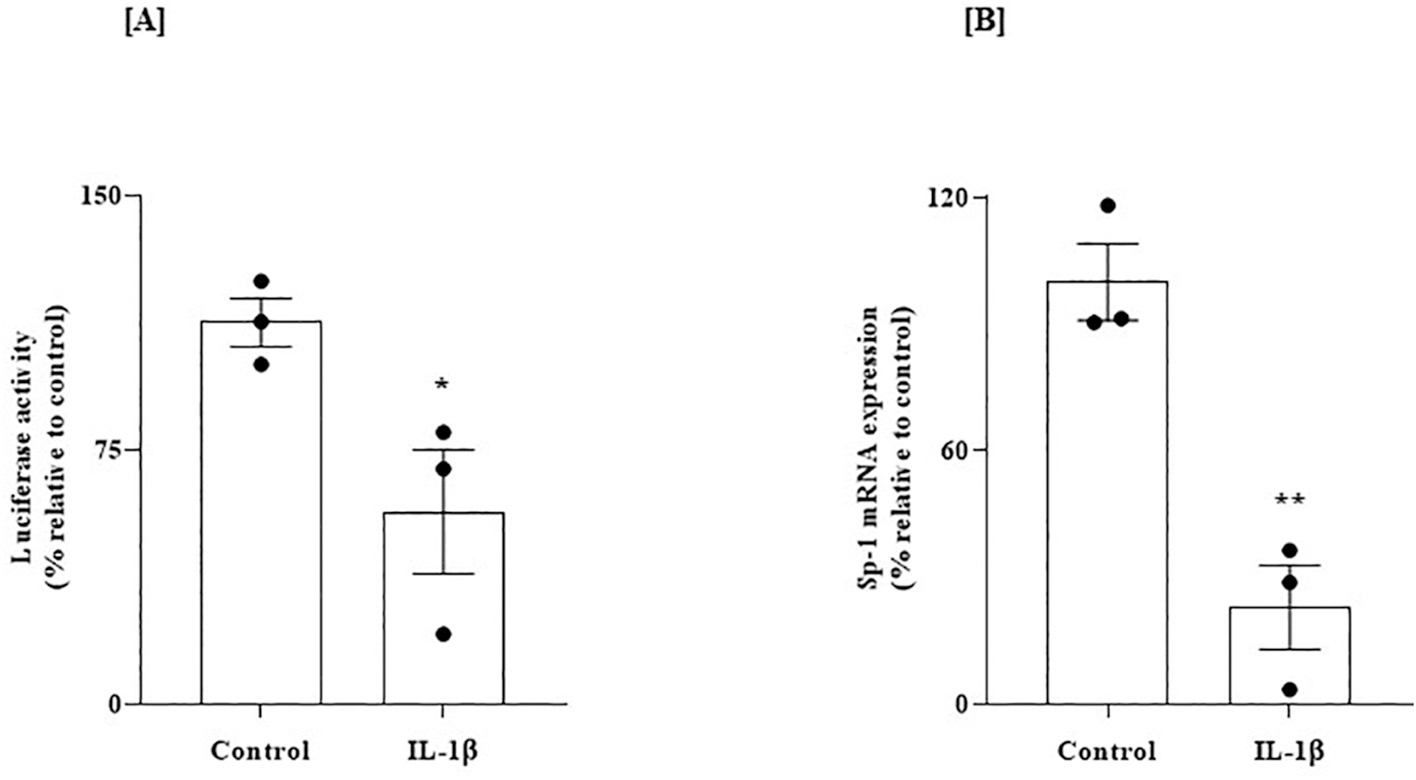
Effect of exposure of SH-SY5Y cells expressing the human *SLC19A2* promoter to IL-1β on (A) promoter activity, and on (B) level of expression of Sp-1 mRNA. (A) Cells transfected with *SLC19A2* minimal-promoter in pGL3 basic vector were exposed to IL-1β (50 ng/mL: 48 h). *SLC19A2* promoter activity (i. e., luciferase activity) was then determined as described previously [48]; data (n = 3) was normalized relative to PGL3 basic vector. (B) Level of expression of Sp-1 mRNA in SH-SY5Y cells treated with IL-1β (50 ng/mL; 48 h) was determined by RT-qPCR; data (n = 3) were normalized relative to β-actin. Statistical significance (*P < 0.05 and **P < 0.01) for luciferase activity and RT-qPCR studies were evaluated by the Student’s *t*-test.

We then examined whether the inhibitory effect of IL-1β on the levels of expression of THTR-1 in SH-SY5Y cells is mediated through *SLC19A2* transcription. SH-SY5Y cells were transfected with human *SLC19A2* minimal promoter (fused to the firefly luciferase reporter), and the effect of exposure to IL-1β (50 ng/mL for 48 h) on promoter activity was determined. We found a statistically significant inhibition in *SLC19A2* promoter activity in cells exposed to IL-1β compared to untreated control cells (*P* < 0.05, Fig. 9A), suggesting the involvement of transcriptional mechanism(s) in mediating (at least part of) the inhibitory effect of IL-1β on thiamin uptake and THTR-1 expression in SH-SY5Y cells.

In another study, we examined the effect of exposure of SH-SY5Y to IL-1β on the level of Sp-1 mRNA expression [this transcription factor plays an important role in driving the activity of the *SLC19A2* promoter; (Anandam et al., 2021; Reidling and Said, 2003)]. Our results showed a statistically significant reduction in the level of expression of Sp-1 mRNA in cells treated with IL-1β compared to untreated controls (*P* < 0.01, Fig. 9B), indicating a potential role for Sp-1 in mediating the inhibitory effect of IL-1β on *SLC19A2* promoter activity in SH-SY5Y cells.

#### 3.3.2. Thiamin availability affects gene expression of genes related to AD in human SH-SY5Y cells

**Fig. 10.**
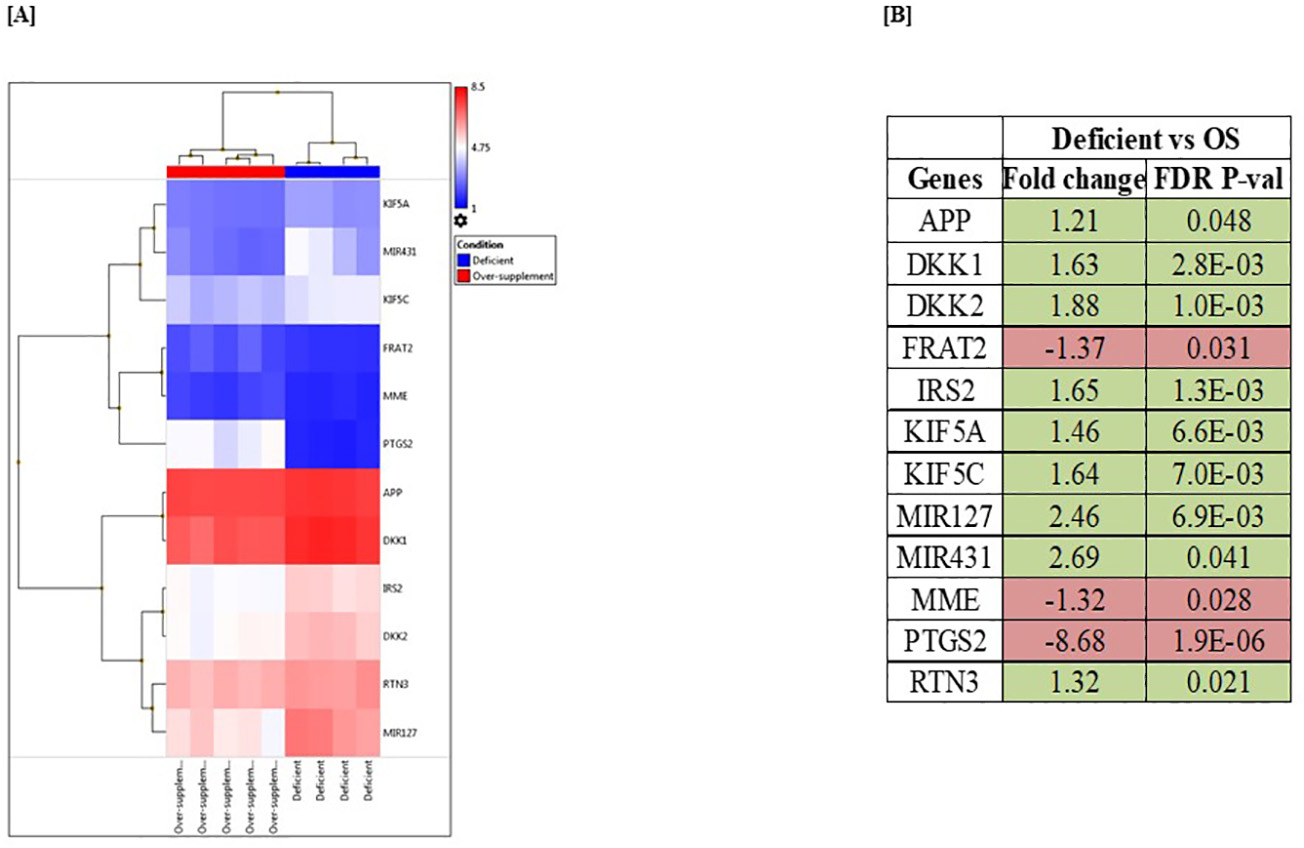
Microarray analysis of SH-SY5Y cells maintained (for 21 days) in thiamin-deficient and over-supplemented growth media. (A) Heatmap of differentially expressed genes associated with Alzheimer’s disease in thiamin-deficient and over-supplemented samples. (B) List of AD-related genes that were significantly altered in response to thiamin availability. Statistical significance of comparison was expressed as FDR P < 0.05 using the Transcriptome Analysis Console. Genes that are up- and down-regulated were highlighted in green and red, respectively.

We lastly examined the effect of thiamin level on gene expression (and ultimately cell physiology) in the human brain SH-SY5Y cells. SH-SY5Y cells were maintained in thiamin-deficient or over-supplemented growth media, and transcriptomic analysis using microarrays was performed. The results showed clear modulations in the gene expression profile when comparing the thiamine-deficient group to the over-supplemented group. A total of 781 genes were differentially expressed with a FDR *P* < 0.05, with 488 being up-regulated and 293 being down-regulated in the thiamine-deficient group (see Supplementary Table S1 for gene expression changes 1.5-fold increase or decrease with a FDR P < 0.05). Interestingly, the list of the differentially expressed genes included many genes associated with putative Alzheimer’s disease pathway (https://www.wikipathways.org/index.php/Pathway:WP2059) (Fig. 10A and B).

## 4. Discussion

Although clear evidence exists suggesting that thiamin homeostasis is altered in patients with AD, that thiamin deficiency aggravates plaque formation, and that many thiamin-dependent processes are diminished in the brains of AD patients (Gibson et al., 2013; Karuppagounder et al., 2009), little is known about possible alteration in the level of expression of THTR-1 and -2 in brain tissue of these patients. Therefore, we used a comprehensive approach, including human, animal, and in vitro studies, to examine the possible molecular mechanisms and whether thiamin transporters (THTR-1 and THTR-2) and neuroinflammation are involved.

Our results revealed that expression of THTR-1 is higher than that of THTR-2 in human and mouse PFC and HIP brain regions. We also found that expression of both THTR-1 and THTR-2 are significantly lower in these brain regions in the AD patients and the 5XFAD model of AD compared to control subjects. To elucidate a potential role of neuroinflammation, we examined the effect of pro-inflammatory cytokines on thiamin uptake by the SH-SY5Y cells, focusing on studying the effect of exposure to IL-1β, IL-6, and TNF-α on thiamin uptake physiology and molecular biology since levels of these pro-inflammatory cytokines have been shown to be elevated in brain regions and cerebrospinal fluid (CSF) of AD patients (Italiani et al., 2018; Veerhuis et al., 1999; Zhao et al., 2003; Zuliani et al., 2007). First, we confirmed that thiamin uptake by these cells is carrier-mediated in nature and that these cells express THTR-1 and THTR-2 (again with an expression of the former being much higher than that of the latter). Our results also showed statistically significant inhibition of carrier-mediated thiamin uptake in cells treated with these cytokines compared to untreated controls. Focusing on IL-1β, we found that the inhibition caused by this cytokine increases as a function of exposure time and that the inhibition is reversible upon removal of IL-1β from the growth medium. In addition, the inhibition in thiamin uptake by IL-1β was associated with a significant decrease in protein and mRNA levels of the predominant THTR-1 (with no change in the level of expression of THTR-2). Since decreased mRNA level of a given gene could be mediated via suppression of the transcription rate of that gene, we tested this possibility by examining the effect of IL-1β on the activity of the *SLC19A2* gene promoter transfected into SH-SY5Y cells. A significant inhibition in the activity of the transfected *SLC19A2* promoter was found in cells treated with IL-1β compared to untreated cells. We also observed a substantial reduction in the level of expression of the nuclear factor Sp-1, which is needed for basal activity of the *SLC19A2* promoter (Reidling and Said, 2003), suggesting the possible involvement of this transcription factor in mediating the inhibitory effect of IL-1β on THTR-1 expression. Thiamin availability was found to affect the expression level of a variety of genes in the human brain SH-SY5Y cells. Our findings using transcriptome analysis of total mRNA prepared from SH-SY5Y cells maintained for 21 days in thiamin-deficient and over-supplemented growth media showed that the expression of 781 genes is significantly altered. Of particular relevance to the aim of this study was the observation that 12 genes that are related to AD pathways were altered. Further investigations are needed to understand the exact relevance of this observation to AD pathophysiology, but some interesting patterns emerged. Dickkopf Wnt signaling pathway inhibitor 1 and 2 (*DKK1* and *DKK2*), each of which can inhibit beta-catenin-dependent Wnt signaling, were both upregulated in the thiamine deficient group – an effect also observed in AD (Vallée and Lecarpentier, 2016). FRAT Regulator Of WNT Signaling Pathway (FRAT2), a positive regulator of the Wnt signaling pathway (Saitoh et al., 2001), was downregulated. Wnt signaling is a requisite for synaptic plasticity and maintenance, and deficient Wnt signaling is associated with aging and AD (Palomer et al., 2019). Insulin Receptor Substrate 2 (*IRS2*), which was upregulated in the thiamine deficient group, is a cytoplasmic signaling molecule that mediates effects of insulin, insulin-like growth factor 1, and other cytokines; *IRS2* is predominant over *IRS1* in the brain, and genetic ablation of *IRS2* in mice reduces amyloid in an AD mouse model (Ochiai et al., 2021). Proteins in the Kinesin heavy chain isoform 5 (KIF5) family mediate the anterograde transport of APP and its processing enzymes, with KIF5A and KIF5C, both of which were upregulated here in the thiamine-deficient group, being exclusively expressed in neurons. Changes in *KIF5A* expression have been inconsistently linked to Alzheimer’s disease (Chen et al., 2021). Reticulon 3 (RTN3), a neuronally-expressed reticulon family protein previously found to negatively regulate BACE1, a protease that is required for the generation of β-amyloid peptides (Aβ) from amyloid precursor protein APP, was also upregulated in the thiamine-deficient group, as was APP itself. RTN3 variants are suggested to be genetic modifiers for RTN3-mediated formation of neuritic plaques in AD (Zou et al., 2018). Two miRNAs (short noncoding RNA molecules) were upregulated in the deficient group – miR-127 and miR-431. Circulating miR-127–3p is a potential biomarker for differential diagnosis in frontotemporal dementia (Piscopo et al., 2018) and according to one meta-analysis, miR-127–3p has the strongest association with AD of any miRNA in the CSF (Takousis et al., 2017). miR-431 regulates axon regeneration in mature sensory neurons by targeting the WNT antagonist Kremen1 (Wu and Murashov, 2013). Axon loss is a hallmark of Alzheimer’s (Coleman and Perry, 2002) and this gene alteration illustrates yet another connection with the Wnt signaling pathway. Membrane metalloendopeptidase (MME), also known as neprilysin (Kato et al., 2022), was downregulated in our thiamine-deficient group. This is striking given that MME is a protease considered among the most important β-amyloid (Aβ)-degrading enzymes with regards to prevention of AD pathology (Miners et al., 2012). Also downregulated in our thiamine-deficient group, *PTGS2* (Prostaglandin-Endoperoxide Synthase 2) encodes COX-2, which produces prostaglandins central to inflammatory and pain responses. Enhanced COX-2 expression is found in AD patients’ brains, and *PTGS2* is suggested to be an AD predisposition gene (Ma et al., 2008).

Accumulating evidence from human and animal studies supports the anti-inflammatory activity of thiamine and its lipid-soluble analog ben fotiamine (Bozic et al., 2015; González-Ortiz et al., 2011; Karabulut et al., 2022; Liu et al., 2017a; Sánchez-Ramírez et al., 2006; Shoeb and Ramana, 2012; Yadav et al., 2010). Correlated with their anti-inflammatory effects, thiamine and benfotiamine supplementations improved learning and memory in different animal models of cognitive deficits (Karabulut et al., 2022; de Moraes et al., 2020). While the poor bioavailability of thiamine has been suggested to limit its clinical benefits in Alzheimer’s disease patients (Blass et al., 1988; Nolan et al., 1991; Rodríguez et al., 2001), its analog benfotiamine has been shown to improve cognitive impairment and reduce amyloid deposition and hyperphosphorylated tau in rodent models of Alzheimer’s disease (Pan et al., 2010; Zhang et al., 2011). Considering the increased recognition of inflammation in the Alzheimer’s brain, our results suggest powerful benefits of high-bioavailability thiamine supplementations as adjunct therapies for managing the pathological alterations and the cognitive symptoms in Alzheimer’s disease.

In summary, our study shows for the first time that the levels of expression of THTR-1 and -2 in AD disease brain are significantly reduced. This may explain the previously reported findings of reduced total thiamin content in the AD brain (Mastrogiacomo et al., 1996). In addition, the results show that exposure of human neuroblastoma cells to pro-inflammatory cytokines leads to a significant suppression in thiamin uptake and expression of THTR-1 and that the effect is transcriptionally mediated. Moreover, the findings point to a role for neuroinflammation in the dysfunction of thiamin transporters (mediated via transcriptional mechanisms) and suggest a beneficial effect of optimizing thiamin levels in the brain of AD patients. The latter can be achieved via administration of pharmacological doses of thiamin [as done in the treatment of conditions like Thiamin-Responsive Megaloblastic Anemia [(an autosomal recessive disorder caused by a mutation in the *SLC19A2*); Lu et al., 2019] to force entry of thiamin into cells via simple diffusion (i.e., to by-pass the need for transport via the cell membrane carrier-mediated process). Since as mentioned earlier that thiamin supplementation is beneficial in reducing plague and tangles as well as in improving memory, at least in experiential animals (Pan et al., 2010; Zhang et al., 2011), together with the documented clinical benefits of such supplementation (Sambon et al., 2021), we believe that supplementation with pharmacological doses of thiamin is reasonable and is of benefit in the treatment of AD and other brain pathological conditions.

## 5. Conclusions

Level of expression of thiamin transporters (THTR-1 and THTR-2) are significantly decreased in brain tissues of AD patients and in animal model of the disease. Also, exposure of neuroblastoma SH-SY5Y cells to pro-inflammatory cytokines leads to inhibition in thiamin uptake via a transcriptionally mediated mechanism. Finally, we found that thiamin availability causes modulation in gene expression profile in SH-SY5Y cells, including those that are relevant to AD pathway.

## Supplementary Material

Supplementary Material

## Figures and Tables

**Table 1:** Sequence of the primers used for RT-qPCR studies.

Gene Name	Forward and Reverse Primer Sequences (5′–3′)

hTHTR-1	GCCAGACCGTCTCCTTGTA; TAGAGAGGGCCCACCACAC
hTHTR-2	TTCCTGGATTTACCCCACTG; GTATGTCCAAACGGGGAAGA
hSp-1	CCATACCCCTTAACCCCG; GAATTTTCACTAATGTTTCCCACC
hβ-actin	CATCCTGCGTCTGGACCT; TAATGTCACGCACGATTTCC
mTHTR-1	GTTCCTCACGCCCTACCTTC; GCATGAACCACGTCACAATC
mTHTR-2	TCATGCAAACAGCTGAGTTCT; CTCCGACAGTAGCTGCTCA
mβ-actin	ATCCTCTTCCTCCCTGGA; TTCATGGATGCCACAGGA

h, human; m, mouse.

## Abbreviations:

AD: Alzheimer’s disease
PFC: prefrontal cortex
HIP: hippocampus
THTR –1 and –2: thiamin transporter –1 and –2
TNF-α: tumor necrosis factor α
IL-1β: interleukin 1β
IL-6: interleukin 6
RT-qPCR: reverse transcription-quantitative polymerase chain reaction
